# Association of fetal hydrocephalus with other embryological anomalies: A prenatal ultrasound-based study

**DOI:** 10.12669/pjms.38.6.5223

**Published:** 2022

**Authors:** Ambreen Surti, Ambreen Usmani, Quratulain Javaid, Sherish Shafique

**Affiliations:** 1 Dr. Ambreen Surti, MBBS, M.Phil-Anatomy Assistant Professor, Department of Anatomy, Bahria University Medical and Dental College (BUM&DC), Karachi, Pakistan; 2 Dr. Ambreen Usmani, MBBS, M.Phil-Anatomy, MCPS-HPE, PGD-Bioethics, PhD- Anatomy Professor Anatomy, Department of Anatomy, Bahria University Medical and Dental College (BUM&DC), Karachi, Pakistan; 3 Dr. Quratulain Javaid, MBBS, PGD-Bioethics, M.Phil Anatomy Assistant Professor, Department of Anatomy, Bahria University Medical and Dental College (BUM&DC), Karachi, Pakistan; 4 Dr. Sehrish Shafique MBBS, FCPS -Medicine, Assistant Professor, Department of Medicine, Bahria University Medical and Dental College (BUM&DC), Karachi, Pakistan

**Keywords:** Congenital anomalies, Fetal hydrocephalus, Prenatal ultrasound, Ventriculomegaly

## Abstract

**Objective:**

To determine the incidence of fetal hydrocephalus in pregnant women and to identify the association of fetal hydrocephalus with other embryological anomalies.

**Methods:**

This comparative cross-sectional study was conducted on 36 pregnant women at a private ultrasound clinic in Karachi over a period of eight months. The participants were divided into age groups, 21-30 years and 31-40 years. Toshiba APLIO 300 ultrasound machine was used to assess fetal age by measuring biparietal diameter (BPD) and femur length, whereas atrium of lateral ventricle was measured to diagnose fetal hydrocephalus.

**Results:**

Twenty-two cases of fetal hydrocephalus were observed in maternal age of 21-30 years with a p-value of 0.011. Severe dilatation of ≥15mm was observed in 85.7% cases in age group of 31-40 years. Cranial anomalies were found in 20 cases with significant results while extracranial anomalies were observed in cases of severe dilatation only. Hydrocephalus was prevalent in male fetuses and was observed in 30 (83.33%) fetuses.

**Conclusion:**

Most cases of fetal hydrocephalus were observed in women of younger age (p=0.011). Fetal hydrocephalus of severe type exhibiting ventricular dilatation >15mm was observed in fetuses of male gender.

## INTRODUCTION

Congenital hydrocephalus which develops in early intrauterine life, is characterized by excessive accumulation of cerebrospinal fluid in the ventricles of the brain that creates excessive pressure on the surrounding neural tissues. The defect is caused by either excessive production of cerebrospinal fluid, decrease rate of absorption or blockade in the passageway of the flow of fluid.^1^ Hydrocephalus in general is considered to have a complex etiology; cerebral hemorrhage, genetic predisposition and brain trauma are common causes.^2^ Systemic review and meta-analysis have documented that the disease is more prevalent in low and middle income countries like Southeast Asia, Latin America and Africa.^3^ Early detection of the defect is crucial as it can lead to cognitive impairments, defects of visual field and cerebral palsy and this can be achieved by modalities like ultrasound, CT scan and MRI. In developing countries ultrasound is the procedure of choice for diagnosis since it is cost effective and serves the purpose without emitting harmful radiation.^4^ Ultrasound, being routinely used in prenatal clinics, Alhassan et al supported the effectiveness of early screening of hydrocephalus.^5^

A Chinese study documented the prevalence of 20.3 per 10,000 births. Out of the total births, 41% had isolated hydrocephalus while others had associated defects like spina bifida.^1^ A study conducted in Ghana has documented 57.3% prevalence of hydrocephalus as compared to other birth defects.^5^ Another research also mentioned the higher prevalence of hydrocephalus in babies with neural tube defects like meningocele, meningomyelocele and encephalocele.^3^,^5^ A Dutch Cohort reported the linkage between aqueduct stenosis and myelomeningocele with development of hydrocephalus.^6^

Another study conducted in Nawabshah, Pakistan showed that the defect was more common in male infants as compared to females with the ratio of 2.5:1.^7^ While a Chinese study documented the presence of congenital hydrocephalus in more than fifty percent of the births with female gender.^1^ It has been postulated that advancing age of the mothers is associated with the development of hydrocephalus. A retrospective study on the fetal autopsy conducted in Tunisia has documented that female who conceive after the age of 35 years have increased risk of giving birth to a child with hydrocephalus. Mothers above the age of forty years are at excessive risk of developing hydrocephalus with lissencephaly at a rate which is thirty-five-fold more than the young mothers.^8^ It was reported that hydrocephalus is more common in babies who have other developmental defects like polygyria, lissencephaly, trisomies, cysts of choroid plexus, holoprosencephaly, hypoplasia of cerebellum and in cases when there is stenosis of aqueduct of Sylvius.

Early detection helps the parents to be mentally prepared to handle such babies, also they have enough time to choose for termination of pregnancy. To our knowledge, no study was previously done in Pakistan that has documented the association of hydrocephalus with advancing age of the mother. Therefore, the study was planned with the objectives to determine the incidence of fetal hydrocephalus in pregnant women and to identify the association of fetal hydrocephalus with other embryological anomalies.

## METHODS

After approval of the study protocol from the Ethical Review Committee of Bahria University Medical and Dental College, Karachi (ERC 47/2018), this comparative cross-sectional study was conducted at a private ultrasound clinic in Karachi over a period of eight months. This research strictly adhered to the codes of Helsinki’s declaration and an informed, written and understood consent was obtained from all participating subjects after explaining the study procedure.

### Study Subjects

Sample size was calculated to be 33 by using open epi, open resource calculator by using population prevalence. However, 36 participants were included by non-probability purposive sampling technique. All pregnant females within the age group of 21-40 years, were divided into two groups, 21 -30 years and 31- 40 years. Both uniparous and multiparous women were part of the study. Women with co-morbidities like essential hypertension and diabetes mellitus and fetuses with any sort of chromosomal anomaly were excluded from the study.

### Imaging Technique

Toshiba APLIO 300 ultrasound machine with a standard convex transducer of 2.5 – 3.5 MHz was used to take all measurements.

### Atrium of Lateral Ventricle (ALV)

Atrium of lateral ventricle is the point where body, posterior and temporal horns of lateral ventricle meet. It was measured after visualization of choroid plexus in coronal plane by taking measurement from inner wall of choroid plexus to the inner wall of ventricle, values >10mm were diagnosed as hydrocephalus. Measurements between 10 -12 mm were diagnosed as mild, while values measuring 12.1-14.9 mm and ≥ 15 mm were diagnosed as moderate and severe forms of hydrocephalus at 21- 39 weeks of gestation.

### Statistical Analysis

Statistical Package for Social Sciences version 23.0 for Windows (SPSS, Inc., Chicago, IL) was used to analyze the data. All quantitative variables were expressed as mean and standard deviation. T-test was used to see the association between fetal hydrocephalus (as measured by measuring the atrium of lateral ventricle in mm) with maternal age and gender. Whereas ANOVA was used to see the association of fetal hydrocephalus with cranial and extracranial anomalies.

## RESULTS

### Presentation of fetal hydrocephalus in maternal age groups

Twenty-two participants were found in age group of 21-30 years and 14 patients of age group 31- 40 years were seen (Table-I). Statistically significant results were observed (p-value = 0.011) for the presence of fetal hydrocephalus (FH) in fetuses of maternal age group of 21 -30 years after applying t-test. (Table-I).

**Table I:** Association of fetal hydrocephalus with maternal age groups.

Hydrocephalus (mm)				

Maternal age	N	Mean	Standard deviation	P value
21-30 years	22	15.28	3.62	0.011*
31- 40 years	14	19.18	3.61	

### Association of fetal hydrocephalus with fetal gender and Measurements of Atrium of Lateral Ventricle

**Fig.1:**
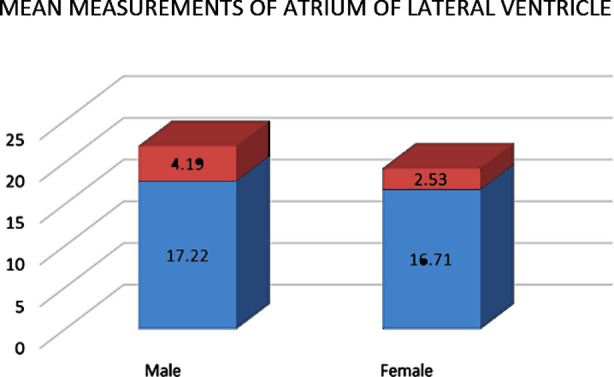
Bar chart showing mean measurements of atrium of lateral ventricle in fetal gender

Thirty cases of FH were observed in male fetuses with a mean measurement of 17.22±4.19 while only 6 cases were observed in female (Fig.1). However, no statistically significant results were observed (p-value 0.780) because of very little difference between the mean measurements of atrium of lateral ventricle.

### Frequency of hydrocephalus based on severity and its association with maternal age

**Fig.2:**
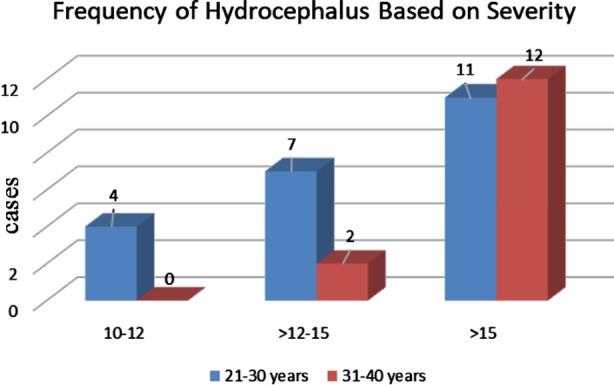
Bar chart showing comparison of frequency of hydrocephalus based on severity.

Based on the measurement of lateral ventricle (LV), FH was classified as mild (10 -12 mm) moderate (12.1 – 14.9mm) and severe (≥ 15mm). 9 Almost 64% cases showed severe dilatation whereas 25% and 11.1% were of moderate and mild types respectively (Fig.2). Severe forms of FH were observed in both 21 – 30 years (11 cases) and 31 -40 years (12 cases) as shown in Fig.2. It was also observed that out of 14 cases of FH in 31-40 years, 85.7% were of severe type and 14.3% were of moderate type, however, no mild dilatation was observed.

### Association of fetal hydrocephalus with cranial and extracranial embryological anomalies

After applying analysis of variance, our study observed FH to be strongly associated with cranial anomalies (20 cases) with a p-value=0.03 (Table-II). Anomalies like spina bifida, meningomyelocele and Dandy Walker syndrome along with extracranial anomalies like fetal ascites, urinary tract malformations, pleural effusion and dilated bowels were associated with severe dilatation of ≥ 15mm (11 cases).

**Table II:** Association of fetal hydrocephalus with the cranial and extracranial embryological anomalies.

	N	Mean	SD	P-value
Isolated hydrocephalus	13	18.48	4.71	0.033*
Cranial anomalies	20	16.06	3.01	
Extracranial anomalies	3	21.76	3.87	

## DISCUSSION

Advancement in science and technology has brought with it an improvement in ultrasonography. This progression along with development in expertise of sonologist has improved diagnosis of FH in 2^nd^ and 3^rd^ trimesters with a higher efficacy.^4^,^10^,^11^

Our study observed prevalence of fetal ventriculomegaly in maternal age group of 21-30 years (p-value = ≤0.011), whereas literature supports the fact that FH is more prevalent in fetuses of mothers with older age group (31-40 years). A study conducted on 241 participants by Chu et al in China observed mean maternal age of 28 years.^12^ In another prospective study conducted in Tripoli on 74 patients, it was observed that 43% patients were in the age group of 20-30 years whereas 62% belonged to age group 30 years or more.^13^ Median maternal age was 25 ± 4.2 years in another study hence corroborating our study results.^14^ In a descriptive cross-sectional study in Pakistan, 35% cases of congenital anomalies were observed in women of age group 26-30 years.^15^ Thereby, observing the results of current study and that of other similar studies younger maternal age-groups are related to congenital anomalies like hydrocephalus in fetuses. Cultural norms where women get married at an early age could be one of the reasons of finding congenital anomalies in fetuses of women with younger age.

We were able to deduce that in our part of the world FH presents in younger age groups, but severity increases with increasing maternal age. The reason for this could be that females are married at a younger age and as it is a more productive age the chances of FH could be more in this age group. The literature, however, has opposite findings where a prospective study on 278 patients showed that 73% had mild dilatation as diagnosed on MRI.^16^ One reason for this contradiction in result could be small sample size and the modality used. MRI being more specific can diagnose mild forms of dilatation more precisely as compared to ultrasound. Chu et al in their study on 241 cases performed scans at 27 weeks of gestation and observed the prevalence of mild ventricular dilatation (65%) where mean maternal age was found to be 28 years.^12^,^14^ Whereas, Breeze et al observed 20 cases of severe dilatation in their study when scans were performed at 28 weeks of gestation with mean maternal age of 19 years.^17^ Reasons for differences could be the younger maternal ages in our study; scans performed at different weeks of gestation; larger sample size and better prenatal care in developed countries where the number of congenital anomalies and its severity can easily be reduced by provision of better health standards and good ante-natal care.

Literature suggests worse prognosis of FH if associated with other anomalies.^16^ Multiple embryological anomalies were observed in our study in association with fetal hydrocephalus. Twenty cases were associated with neural tube defects like spina bifida and meningomyelocele. Few cases of Dandy walker syndrome, dangling choroid, dilated 3^rd^ and 4^th^ ventricles and cerebral aqueduct dilatation were also identified. Isolated cases, without any other congenital anomaly, were few followed by only three cases of extracranial anomalies. Chu et al in their study found 66% cases of FH associated mostly with cranial anomalies, mostly exhibiting mild dilatation (70.9%).^12^ Several studies have documented the association of FH with neural tube defects like spina bifida.^1^,^3^,^5^,^6^,^17^ Literature and previously conducted studies observed 10%-70.9% association of FH with other anomalies and have shown a 41-76% increase in association of anomalies with the increase in atrial width measurements.^15^ Etchegaray et al. observed 62.7% association of FH with other anomalies where 50% were associated with mild dilatation while 64% with severe dilatation, which is similar to our study. However, the authors did not find any difference in terms of anomalies in the three groups.^18^ One reason for this difference could be the possibility of difference in the populations and secondly improved and more advanced use of technology in developed countries. Forty-three cases of hydrocephalus were found to be associated with stenosis of aqueduct of Sylvius and demonstrated enlargement of 3^rd^ ventricle and abnormalities of corpus callosum in yet another research.^19^ Hence further strengthening the results of our study. The results of our study and that of others prove that hydrocephalus is found to be mostly associated with neural tube defects like spina bifida and meningomyelocele.

As documented in literature, our study also observed a male predilection of FH.^1^,^7^,^14^,^20^ Similar findings were observed in a retrospective study conducted on 44 cases in 2018 where 26 cases of FH were of male fetuses.^21^

### Limitations of the study

Due to small sample size and being single center oriented, the results cannot be generalized.

## CONCLUSION

Male fetuses born to mothers in younger age group 21-30 years exhibited a predilection for fetal hydrocephalus. Fetal hydrocephalus classified as mild, moderate and severe forms, was found to be more prevalent in fetuses of younger mothers and was found to be associated with numerous other cranial and extracranial embryological anomalies. Fetal hydrocephalus which can be classified into mild, moderate and severe forms on the basis of atrial width measurements can present either isolated or associated with cranial and extracranial embryological anomalies.

### Author`s Contribution:

**AS** conceived, designed and prepared the manuscript. She is also responsible for the integrity and accuracy of the study.

**AU** editing, review and proof reading.

**QJ** manuscript writing and editing.

**SS** literature search and editing.
